# Successful survival, growth, and reproductive potential of quagga mussels in low calcium lake water: is there uncertainty of establishment risk?

**DOI:** 10.7717/peerj.1276

**Published:** 2015-11-03

**Authors:** Clinton J. Davis, Emma K. Ruhmann, Kumud Acharya, Sudeep Chandra, Christopher L. Jerde

**Affiliations:** 1 Department of Biology, University of Nevada, Reno, NV, United States; 2 Department of Water Resource Management, University of Nevada, Las Vegas, NV, United States; 3 Division of Hydrologic Sciences, Desert Research Institute, Las Vegas, NV, United States

**Keywords:** Quagga mussel, Establishment risk, Calcium, Lakes, Life history

## Abstract

The risk of quagga mussel (*Dreissena rostriformis bugensis* Andrusov 1897) establishment into water-bodies of the western US has expanded the geographic concern regarding the ecological and economic impacts this species will have in aquatic ecosystems. Thresholds based on calcium concentrations, an element critical for mussel growth and physiology, have been used as a primary predictor of quagga mussel establishment success to aid management decisions. We evaluated the invasion potential of quagga mussels in low calcium waters using laboratory experiments to compare the survival, growth and reproductive potential of adult mussels held for 90 days at low (9 and 12 ppm), moderate (15 to 32 ppm) and high (72 ppm) calcium water concentrations. In conjunction with adult experiments, veliger stage survival, growth and settlement were evaluated under similar low, moderate, and high calcium water treatments. Adult mussels survived, grew and showed reproductive potential in low calcium water (12 ppm). Veligers were also able to survive, grow and settle in low calcium water. Higher levels of natural seston biomass appeared to improve adult mussel life history performance in low calcium water. Survival curve analysis predicted that 99% adult mortality could occur in <170 days at 9 ppm and 12 ppm, however water with >15 ppm could have adults surviving more than a year. The results from these bioassays provide further evidence that quagga mussels have higher risk of establishment in low calcium lakes if habitats exist that have slightly elevated calcium. These results should help emphasize the vulnerability of water-body in the 12 to 15 ppm calcium range that could potentially be at risk of establishing sustainable quagga mussel populations. Furthermore, these results provide insights into the uncertainty of using a single parameter in assigning establishment risk given the complexity of variables in specific water-bodies that influence life history performance of introduced species.

## Introduction

The establishment and spread of dreissenid mussels exemplifies how disruptive invasive species can be via ecological impacts to foodwebs (Strayer, Hattala & Kahnle, 2004), nutrient cycling (Hecky et al., 2004) and biodiversity (Karatayev, Burlakova & Padilla, 1997). The presence of these invasive mussels have the capacity to significantly alter the existing ecosystem patterns and processes that not only have numerous consequences for the ecological integrity but the overall natural economic and recreational benefits of invaded water-bodies (Nalepa & Schloesser, 2013). Due to the recent spread of dreissenid mussels to western United States (Nalepa, 2008), management resources have increased with a major focus on the prevention of this species (e.g., $930,000 allocated to US Fish and Wildlife 2013 budget “Quagga Mussel Containment in the West”). Information used to forecast dreissenid establishment in the western United States is based largely on knowledge developed during the dreissenid expansion in the Great Lakes Basin and Europe (Mackie & Claudi, 2013). It is uncertain how well the synthesis of these regional dreissenid studies that are largely based on zebra mussel ecology, applies to water-bodies of the western United States that are recently at risk of quagga mussels invasion.

Predicting which water-bodies have the highest risk of invasion will help channel limited management resources to the systems most vulnerable to supporting sustained populations of quagga mussels. Several water quality parameters that have a range of certainty regarding effects on dreissenid life history include temperature, dissolved oxygen, pH, salinity, turbidity and calcium (Mills et al., 1996; Wright et al., 1996; Karatayev, Burlakova & Padilla, 1998; Thorp, Alexander & Cobbs, 2002; Jones & Ricciardi, 2005; Claudi et al., 2012). In particular, quagga mussel tolerance for cold and warm temperatures, combined with apparent flexibility in diet regimes has been cited as a likely reason for their displacement of zebra mussels in the Great Lakes Basin (Baldwin et al., 2003) and expansion to the southwestern United States (Nalepa, 2008). Uncertainty exists regarding quagga tolerance to low calcium conditions (Cohen, 2007) as, to-date, low calcium tolerance thresholds are based on field observations of presence or absence (Jones & Ricciardi, 2005) with only a few experimental studies in the northeastern United States (Frischer et al., 2005; Baldwin et al., 2012). Observational studies attribute the establishment of dreissenids in low calcium water-bodies to localized “hotspots” of calcium (e.g., concrete boat ramps and piers, river inflows, groundwater) and, in many cases, appear to have extremely limited recruitment success (Lake George, Wimbush et al., 2009) or are sink populations that are dependent on larval production outside of the system (e.g., Illinois River, Schneider et al., 2003).

There are several stages and processes of dreissenid life history that are critical to evaluate when assessing their establishment potential in an aquatic system. Since both sessile adults and planktonic larval veligers can be spread to new locations by anthropogenic (e.g., boat hulls) and natural (e.g., downstream water flow) processes, the survival and growth of these stages need to be examined. Successful development of reproductive cycles (e.g., gametogenesis, fertilization) needs to occur in the colonized systems or the resulting population will be a “sink” rather than a “source” (Horvath et al., 1996). Sink populations can also develop if fertilized eggs fail to developed into zygotes, zygotes are unsuccessfully develop into veligers and if planktonic veligers are not able to progress into the juvenile sessile mussel stage during the process of settlement. These quagga mussel characteristics represent life history stage that need to be assessed in order to assess establishment risk. Experiments are needed that examine the performance of quagga mussels in various life history stages using water from western United States that represent the ambient water quality and food quality/quantity in those systems.

The objectives of this study were to quantify the life history performance of mussels held under laboratory conditions for an extended time period in waters from a lake in the Western United States where quagga mussels are considered to have a low risk of invasion (e.g., Lake Tahoe) in comparison to (1) water from Lake Mead where the mussels have established (positive control) and (2) calcium-amended treatments from the low calcium lake. We hypothesized that mussels reared in low calcium water versus calcium amended and positive control waters would have (1) lower adult and veliger survival rates, (2) lower adult and veliger growth rates, (3) reduced production of viable gametes in adults, and (4) reduced veliger settlement rates. The capability of quagga mussel adults and veligers to complete these vital biological processes in low-calcium waters would provide empirical evidence that management agencies should also consider the threat from quagga mussel even in water-bodies with calcium concentrations previously believed to be intolerable to quagga mussel establishment (Whittier et al., 2008).

## Materials and Methods

### Experimental water quality conditions

Mussel survival, growth, settlement, and reproductive potential in Lake Tahoe waters representative of different calcium and trophic conditions were evaluated as experimental treatments. Water was collected from Tahoe Keys, CA (38°55′45″N, 120°0′51″W) a network of shallow channels connected to Lake Tahoe and Lake Tahoe at Cave Rock, NV (39°2′40″N, 119°56′56″W). These two locations represent contrasting trophic conditions that occur in Lake Tahoe and represent low calcium concentrations similar to many mountain lakes in the western United States (USEPA, 2009). Tahoe Keys water was also amended with calcium chloride to represent near-shore and benthic habitats (e.g., sediment interstitial water, Asian clam beds) with elevated calcium concentrations (15–32 ppm Ca). These waters were amended to mimic the conditions of water found at the bottom of lake where invasive Asian clams (*Corbicula fluminea*) have established. Water from Lake Mead, where the adult and veliger mussels were collected for life history experiments, was a positive control treatment to compare to experimental treatments using Lake Tahoe water (Table 1). Discrete water quality parameters (dissolved oxygen, temperature, and conductivity) were documented at the field sites during water collections. A 1 L water sample was collected during water collection and subsampled for analysis of ion chemistry (calcium, magnesium, potassium, sodium), pH and algal biomass (chlorophyll *a*). Ion chemistry was analyzed using an inductively coupled plasma mass spectrometer (Agilent Technolgies 7500ce), pH was measured using a benchtop meter (Daigger 5500) and pheophytin corrected chlorophyll *a* determined fluorometrically (Strickland & Parsons, 1972) using a Turner 10-AU fluorometer after 24 h extraction in methanol (Marker, Crowther & Gunn, 1980).

**Table 1:** Experimental replication for each treatment assessed for the evaluated life history metrics of quagga mussels.

	Positive control	Low calcium		Tahoe Keys calcium amended					
Life history metric	Mead	Tahoe Cave Rock	Tahoe keys	Tahoe Ca amended 15 ppm	Tahoe Ca amended 21 ppm	Tahoe Ca amended 24 ppm	Tahoe Ca amended 29 ppm	Tahoe Ca amended 32 ppm	Tahoe Ca amended 34 ppm
Survival									
Adult	20	20	20	20	20	20		20	
Veliger	20		20		20		20		20
Growth									
Adult	19	10	16	19	18	16		18	
Veliger	4		2		3		3		4
Adult gamete production	18	9	16	19	18	16		18	
Veliger settlement	2		2					1	

### Adult experiments

Adult quagga mussels were collected from Lake Mead, NV-AZ in Callville Bay (36°8′26.″N, 114°43′7″W) in March using a petite Ponar. Gravel with attached mussels was transported in aerated 20-liter containers to a quarantined refrigeration unit. Similar sized mussels (9.2 ± 1.1 mm) were removed from substrate, placed in 9.5-liter containers and acclimated for 48 h at 20 °C in Lake Mead water before placing individuals into experimental treatments. Twenty replicates for Mead (positive control), Tahoe Cave Rock (low calcium), Tahoe Keys (low calcium) and Tahoe Keys calcium amended (Tahoe Ca amended 15 ppm, 20 ppm, 25 ppm, 32 ppm) treatments consisted of a single mussel placed in a 9.5 L pail filled with 7 L of treatment water. Containers of mussels were quarantined in a walk-in refrigeration unit set at 19 ± 2 °C which is the mean summer temperature in the epilimnion of Lake Tahoe.

Every two days the water quality conditions in containers and survival status of adults were assessed. A YSI-85 was used to monitor dissolved oxygen, temperature, and specific conductivity in individual containers. Live or dead status of mussels was determined by noting gape and response to touch. Flesh from dead mussels was removed, placed in 10% buffered formalin for 24 h and fixed with 70% ethanol for reproduction analysis. Fresh treatment water was added to each pail by dumping and refilling 50% of the water volume.

Mussel growth was estimated using changes in length and mass from the beginning to the end of the 90 day experimental period. Changes in length were determined by measuring shell length at the beginning and end with digital calibers. Wet mass of whole mussels was also measured to include changes in soft tissue plus shell mass. Growth rate (week^−1^) for adults was calculated using changes in mass according to the formula:

\begin{eqnarray*} \mu =\frac{\ln {M}_{f}-\ln {M}_{i}}{t} \end{eqnarray*}

where *M_i_* and *M_f_* were the initial and final mass (mg) and *t* is the duration of the experiments in weeks. Preserved soft tissue was placed in paraffin embedding cassettes and processed for histological examination by producing hematoxylin and eosin-stained 5 µm tissue sections permanently mounted on slides. Reproductive potential was based on bright-field microscopy (40 ×–200 × magnification) of permanent mounts to identify gametes (sperm or egg). Presence and type of gametes determined mussel sex (male or female), while gamete absence yielded indeterminate sex.

### Veliger experiments

Culturing conditions for veliger bioassays were tested prior to experiments and it was determined that, regardless of water type, veliger health begins to deteriorate after 28 days in a laboratory setting. Survival, growth and settlement experiments were limited to 28 to 30 days to lessen effects of laboratory conditions while maximizing the experimental period.

Umbo veligers were collected using a 63 µm plankton net from Lake Mead at the Callville Bay Marina in August via horizontal tows at 12 m. Samples were kept chilled during transport to the lab where they were settled in beakers for one hour before using cross-polarized microscopy (Nikon SMZ 1000 with 10× magnification) to select experimental organisms. Veligers that were in the umbonal stage, visibly active (e.g., cilia movement, foot extension, swimming, internal organ movement), and had large shell areas (0.037 ± 0.011 mm^2^) were pipetted into petri dishes.

Five replicate containers for the positive control (Mead), low calcium (Tahoe Keys) and Tahoe Keys calcium amended (Tahoe Ca amended 21 ppm, 29 ppm, 34 ppm) treatments consisted of petri dishes with 5 veligers in each dish filled with 45 mL of treatment water. Water used for treatments in veliger experiments was filtered through a 35 µm Nitex mesh to remove zooplankton. Experimental treatments were kept in an incubator at 19 °C under a light:dark cycle of 8:16 h. Observations every 2 to 3 days assessed survival status based on visible signs of activity (e.g., cilia movement, foot extension, swimming) or mortality (e.g., empty shell, decomposition). Fresh treatment water was added to each dish by exchanging 50% of water volume every 3 days. Ammonia and pH in petri dish water was tested using test strips during exchanges to ensure stable water quality. Digital images for each veliger (5 per treatment replicate) were documented on day 0 and 28 at 80× magnification to estimate growth rates based on the average change in shell area. Shell area calculated using Image J (1.47v Java 1.6.0_20, National Institutes of Health, USA) allowed growth rate (week^−1^) estimates according to the forumula:

\begin{eqnarray*} \mu =\frac{\ln {A}_{f}-\ln {A}_{i}}{t} \end{eqnarray*}

where *A_i_* and *A_f_* were the initial and final average veliger shell area (μm^2^) and *t* isthe duration of the experiments in weeks. Growth rates of surviving veligers are reported as the average ± standard deviation for each treatment.

Veliger settlement experiments compared three treatments (Mead, Tahoe Keys, and Keys 32 ppm) over a period of 30 days. Umbo veligers were collected in mid-July from the same location and using same procedures as the growth/survival experiments. Samples were settled in cones for 2 h to concentrate veligers at the bottom while swimming predators (e.g., copepods) remained in suspension. Approximately 3,500 veligers were added to each treatment tank (16 L) by adding 14 mL of concentrated aliquots (*ca.* 250 veligers mL^−1^). The bottom of each tank was completely covered with glass tiles (25 cm^2^). Tanks were kept at 20 °C, aerated, and under a light:dark cycle of 12:12 h. Fresh treatment water was added every three days to compensate for evaporative loss. Tiles were removed at 30 d, rinsed lightly, and the number of attached plantigrades were counted using cross-polarized microscopy. The total number of veligers in each tank used to estimate settlement rates (i.e., number settled veligers / total veligers in tank) was corrected using survivorship data from growth/survival experiments (e.g., 12 ppm = 30% survival).

### Statistical analysis

Adult and veliger survival was analyzed using logistic regressions to test whether survival rates differed among calcium treatment waters. Analysis of variance (ANOVA) followed by Tukey’s multiple comparison tests was used to test the overall effect of water treatments on adult growth rates. Veliger growth rates were analyzed using Wilcoxon signed-rank tests due to non-normality of data. Replicates for data analysis (Table 1) included (1) survivorship of 20 individual adults/veligers, (2) growth rates based on 10–19 surviving adults and 2–4 replicate containers that had surviving veligers, (3) adult gamete production from surviving adults (minus 1 replicate damaged from Tahoe Cave Rock and Mead), and (4) veliger settlement based on total number of settled and attached larvae in 1–2 tanks. All statistical approaches were completed using R v3.1.2 (R Core Team, 2013).

Another approach to evaluating survival is through time to event analysis of the mortality of individuals (Cox, 1962; Hosmer & Lemeshow, 1999) under various calcium concentrations. Time to event analysis requires regular monitoring of the fate of individuals so only adult mussel survival was analyzed. To assess the survival of adult mussels, we fit the Gompertz distribution survival function (Gompertz, 1825). The probability density function for the Gompertz distribution is,

\begin{eqnarray*} \Pr(T=t)={e}^{t \lambda +(1-{e}^{t \lambda })\xi }\lambda \xi , \end{eqnarray*}

where the parameter *λ* > 0 is a scale parameter and *ξ* > 0 is a shape parameter for the random variable of time *T*(*t* ≥ 0). This flexible continuous probability distribution allows for survival modeling of time to event data. For completeness and comparison, we also provide the Kaplan–Meier estimates with 95% confidence bands, and we tested for differences between the survival rates of different calcium concentrations using a log-rank test (Hosmer & Lemeshow, 1999). We chose to make six comparisons using the log-rank test: 9 ppm–12 ppm, 12 ppm–15 ppm, 15 ppm–21 ppm, 21 ppm–24 ppm, 24 ppm–32 ppm, 32 ppm–72 ppm. Setting a significance level of 0.05 and having 6 multiple comparisons results in a Bonferroni adjusted *p*-value of 0.0083 necessary to conclude a significant difference between a pair. All calculations were conducted in Mathematica (Wolfram Research, Inc., Version 9.0.1.0 Champaign, IL, 2014).

## Results

### Water quality conditions

Most water quality variables in treatment waters remained similar to field site values during the experimental period (Table 2). Relatively stable measures of temperature (19–20 °C), dissolved oxygen (7–8 mg L^−1^), and pH (7.0–8.4) were recorded in experiments. Concentrations of magnesium (2.05–21.6), potassium (1.20–3.89 ppm), and sodium (6.34–77.1 ppm) in ambient waters and calcium-amended treatments were comparable to field conditions of source water throughout the experiment. Additions of calcium chloride elevated the specific conductivity of treatments (165–244 μS cm^−1^) above ambient waters from Tahoe (92–143 µS cm^−1^). Concentrations of chlorophyll *a* averaged from 4.2 to 26 *μ*g L^−1^ from field locations and decreased in all source barrels for treatments (1.2–8.4 *μ*g L^−1^) during the holding period.

**Table 2:** Water quality conditions for treatment waters sampled from field locations, stored in lab, and in containers holding adults or veligers.

Treatment water	pH	Temperature (°C)	Dissolved oxygen (mg L^−1^)	Specific conductivity (μS cm^−1^)	Calcium (ppm)	Sodium (ppm)	Potassium (ppm)	Magnesium (ppm)	Chlorophyll a (μg L^−1^)
Tahoe Cave Rock									
Field	7.9 ± 0.21	10 ± 3	9 ± 1	95 ± 18	8.88 ± 0.71	6.34 ± 0.64	1.52 ± 0.16	2.05 ± 0.22	4.2 ± 1.8
Lab		19 ± 1	8 ± 1	92 ± 3	8.87 ± 0.55	6.41 ± 0.20	1.58 ± 0.04	2.09 ± 0.04	1.8 ± 1.4
Adult^a^		20–20	8–8	90–87					
Tahoe Keys									
Field	8.4 ± 0.56	14 ± 4	10 ± 1	140 ± 12	12.1 ± 2.57	7.26 ± 0.83	1.35 ± 0.13	3.64 ± 0.44	26 ± 14
Lab	8.1 ± 0.58	19 ± 1	7 ± 1	143 ± 12	12.6 ± 0.66	7.73 ± 0.07	1.20 ± 0.06	3.75 ± 0.04	
Adult^a^		19–19	8–7	147–141					3.9 ± 0.7
Veliger	7.5	19			12.9 ± 0.46				
Tahoe Ca amended 15 ppm									
Lab	8.2 ± 0.65	19 ± 1	7 ± 1	165 ± 13	15.4 ± 2.83	7.18 ± 0.77	1.32 ± 0.14	3.65 ± 0.39	8.4
Adult^a^		19–19	7–7	167–164					7.1 ± 5.8
Tahoe Ca amended 21 ppm									
Lab	8.2 ± 0.67	19 ± 1	7 ± 1	206 ± 13	20.9 ± 3.41	7.18 ± 0.86	1.29 ± 0.14	3.62 ± 0.40	5.5
Adult^a^		19–19	7–8	208–205					6.0 ± 2.9
Veliger	7.5	19			21.0				
Tahoe Ca amended 24 ppm									
Lab		19 ± 2	7 ± 1	194 ± 27	24.4 ± 3.87	7.27 ± 0.60	1.47 ± 0.11	2.60 ± 0.34	3.9 ± 3.8
Adult^a^		20–20	7–8	203–181					
Tahoe Ca amended 29 ppm									
Veligers	7.0	19			29.3				
Tahoe Ca amended 32 ppm									
Lab		19 ± 2	7 ± 1	239 ± 23	32.0 ± 4.58	7.24 ± 0.77	1.45 ± 0.17	2.60 ± 0.52	8.1 ± 8.5
Adult^a^		20–20	7–7	244–235					
Tahoe Ca amended 34 ppm									
Veliger	7.5	19			34.0				
Mead									
Field		17			72.0	65.9	3.19	18.2	5.9
Lab	8.3 ± 0.01	19 ± 1	7 ± 1	857 ± 36	71.8 ± 3.17	77.1 ± 4.59	3.89 ± 0.21	21.6 ± 1.04	1.2 ± 1.1
Adult^a^		20–20	7–7	866–860					
Veliger	8.0	19			69.0				

**Notes.**

a Reported as a range (Max–Min) or mean ± SD.

### Adult and veliger survival and growth rates

**Figure 1:**
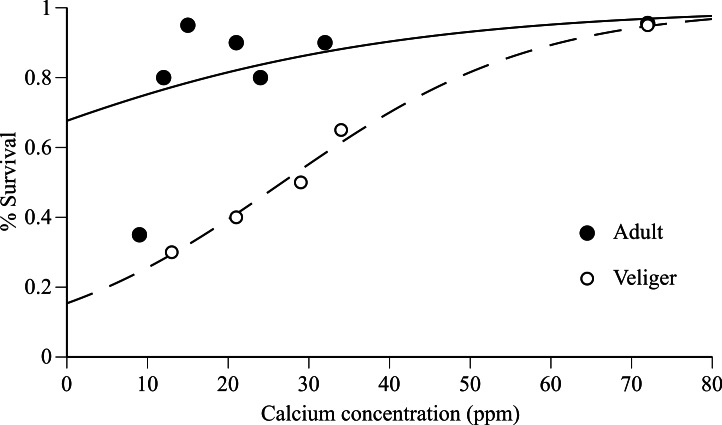
Adult and veliger quagga mussel survival versus calcium concentration in experimental water. Survival of adults (after 90 days) and veligers (after 30 days) versus calcium concentration in experimental water from Tahoe Cave Rock (9 ppm), Tahoe Keys (12 ppm), Tahoe Ca amended water (15–34 ppm) and Mead (72 ppm). Logistic regression model fit to data shown for adult (solid line) and veliger (dashed line).

Adult survival after 90 days was extremely high (80–95%) in Tahoe Keys (12 ppm Ca), Tahoe Keys calcium amended (15, 21, 24, 32 ppm Ca), and Mead (72 ppm Ca) treatments but substantially lower (30%) in Tahoe Cave Rock (9 ppm Ca) (Fig. 1). Veliger survival increased gradually with increasing calcium (30–95%). Logistic regression results (Fig. 1) provide evidence that increases in calcium improves mussel survivorship, though the survivorship increase was especially significant in the larval stage (*P* < 0.001, Chi-square test).

**Figure 2:**
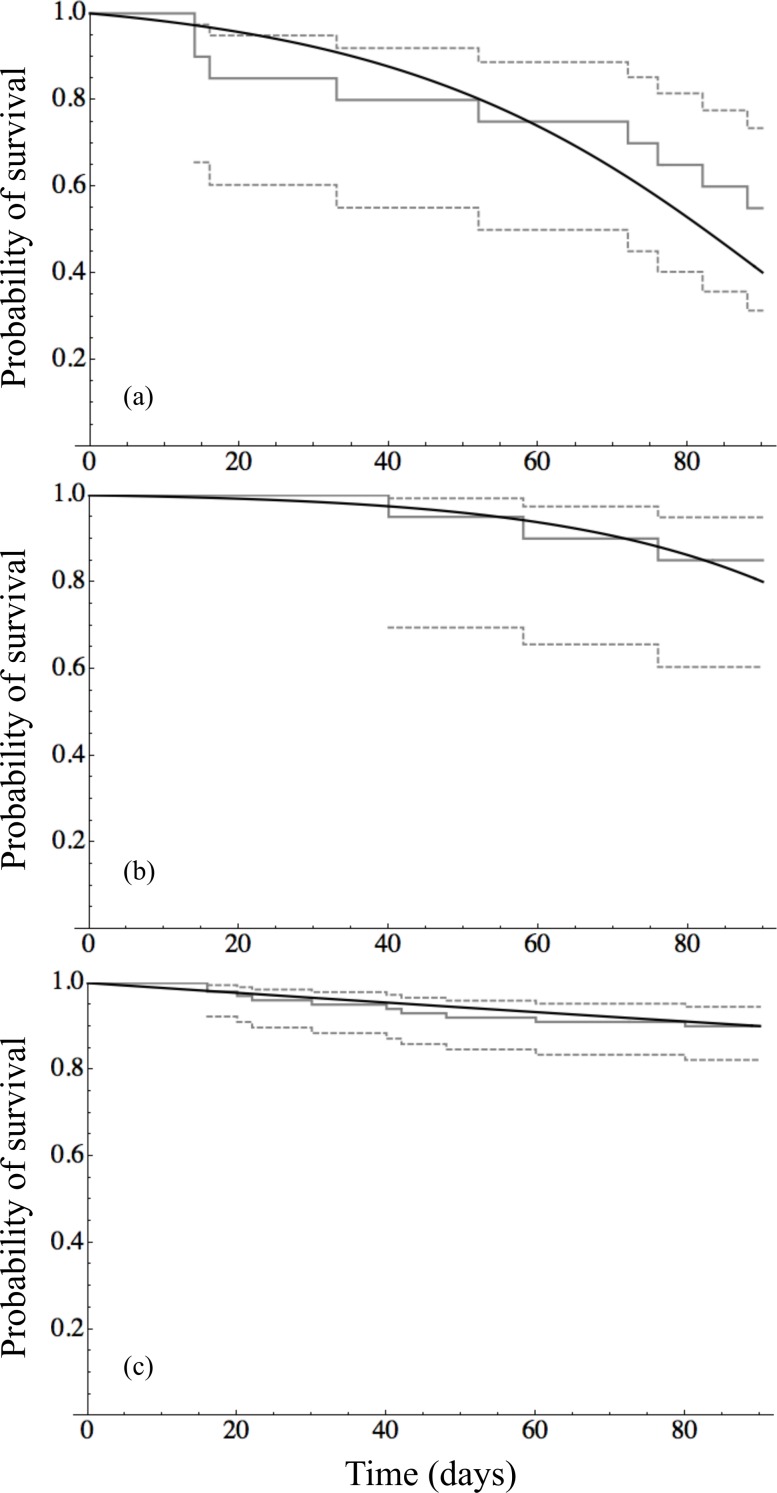
Survival analysis for adult mussels. (A) Tahoe Cave Rock (9 ppm calcium) (*n* = 20), (B) Tahoe Keys (12 ppm calcium) (*n* = 20), and (C) Tahoe Keys Ca amended (15, 21, 24, 32 ppm calcium) and Mead (72 ppm calcium) (*n* = 100). The black line is the continuous function of the Gompertz survival distribution. The solid gray line is the Kaplan–Meier estimate survival function with the dashed gray lines representing with 95% confidence bands of the Kaplan–Meir estimate.

Time to event analysis of the mortality of individuals revealed that survival of adult mussels differed significantly between only two groups: 9 ppm–12 ppm (*p*-value < 0.0001) and 12 ppm–15 ppm (*p*-value = 0.005). Panels in Fig. 2 show the survival curves for the three unique survival curves 9 ppm (*n* = 20), 12 ppm (*n* = 20), and 15–72 ppm (*n* = 100) combined. The Gompertz survival distribution shows a step decline in survival of adults in the 9 ppm Ca treatment nearing the end of the observational period at 90 days. In contrast, adult survival for 12 ppm Ca and in the combined group of 12–72 ppm Ca show considerable survival after 90 days. Table 3 provides the Maximum Likelihood Estimates of the parameters for the Gompertz survival distribution and the projected number of days to achieve 50%, 90%, and 99% mortality. Although the projected number of days of 90% and 99% mortality are well beyond the 90 day observational window of this study, they do indicate that at low calcium concentrations (9 ppm and 12 ppm) there is the possibility of near complete mortality leading to possible extinction of adult quagga mussels in <170 days.

**Table 3:** Parameters of the Gompertz survival distribution used to calculate the estimated number of days (*t*) to reach 50%, 90%, and 99% mortality of adult mussels.

Treatment Ca concentration	Parameters		*t*_50%_	*t*_90%_	*t*_99%_
	*λ*	*ξ*			
9 ppm	0.034	0.046	82	116^a^	136^a^
12 ppm	0.039	0.0069	119^a^	149^a^	167^a^
15–72 ppm	0.0003	4.34	>365^b^	>365^b^	>365^b^

**Notes.**

a Indicates projected survival beyond the duration (90 days) of this study.

b Indicates projected survival beyond one year.

**Figure 3:**
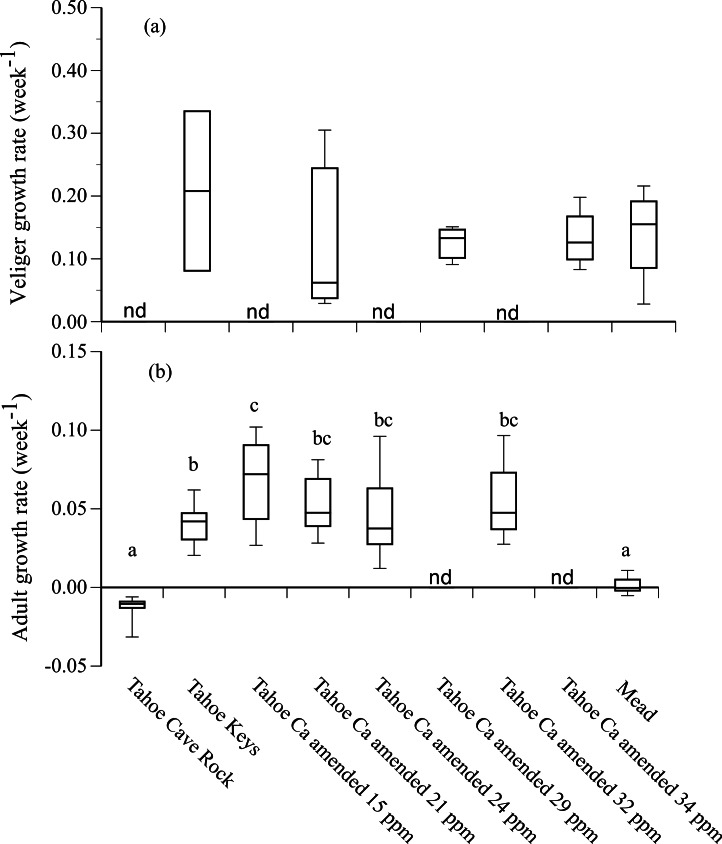
Adult and veliger growth rates in experimental treatment water. Growth rate (week^−1^) of (A) veliger and (B) adult quagga mussels in experimental treatment water from Lake Tahoe and Lake Mead. Lower case letters (a, b, c) indicate significant differences among treatments based on results from Tukey’s multiple comparison tests for adult growth rates. Growth rates for some treatments were not determined (nd).

Growth rates of adult mussels that survived 90 d were positive in Tahoe Keys and Tahoe Keys calcium amended treatments (mean = 0.04–0.07 wk^−1^) and negative in Tahoe Cave Rock (mean = − 0.01 wk^−1^) (Fig. 3). Adults in Mead water exhibited negligible growth. Statistical analysis (ANOVA, Tukey’s multiple comparison) confirmed adult growth rates were significantly different for (1) Tahoe Cave Rock and Mead compared to Tahoe Keys and Tahoe Keys calcium amended treatments (*P* < 0.0001) and (2) Tahoe Keys compared to Tahoe Keys calcium amended 15 ppm (*P* = 0.006). Veligers that survived also showed substantial but variable growth over 28 d in Tahoe Keys, Tahoe Keys calcium amended, and Mead treatments (mean = 0.13–0.21 wk^−1^). Statistical comparisons (Wilcoxon signed-rank) of veliger growth rates among treatments were not significantly different.

### Adult reproductive potential

Analysis of gametogenesis for adults that survived 90 d revealed a high percentage (88–95%) of reproductively viable mussels in Tahoe Keys, Tahoe Keys calcium amended treatments and Mead compared to relatively low viability (11%) in Tahoe Cave Rock (Table 4). However, adults with gametes were relatively low (28%) in Mead water. Pre-treatment adults (i.e., adults from the population at the beginning of the experiments) that were largely of indeterminate sex showed a low percentage (11%) of reproductively-viable individuals, ensuring that gametogenesis primarily occurred during the 90 d experimental period.

**Table 4:** Adult mussel sex and percent reproductively viable from before the experiment (pre-treatment) and from mussels in each treatment that survived the 90-day experiments.

Treatment	Sex	Count	% Reproductively viable^a^
Pre-treatment	Male	2	11%
	Female	0	
	Indeterminate	18	
Tahoe Cave Rock	Male	0	11%
	Female	1	
	Indeterminate	8	
Tahoe keys	Male	7	88%
	Female	7	
	Indeterminate	2	
Tahoe Ca amended 15 ppm	Male	12	95%
	Female	6	
	Indeterminate	1	
Tahoe Ca amended 21 ppm	Male	9	100%
	Female	9	
	Indeterminate	0	
Tahoe Ca amended 24 ppm	Male	8	94%
	Female	7	
	Indeterminate	1	
Tahoe Ca amended 32 ppm	Male	11	100%
	Female	7	
	Indeterminate	0	
Mead	Male	5	28%
	Female	0	
	Indeterminate	13	

**Notes.**

a % reproductively viable = (male count + female count) / (male count + femaile count + indeterminate count).

### Veliger settlement potential

Veligers settled after 30 d in all treatment waters tested. The number of settled veligers increased with higher calcium concentrations (40 settled in Tahoe Keys, 163 settled in Tahoe Ca amended 32 ppm, 361 settled in Mead water), as did the corresponding settlement rates (4%, 9%, 11% respectively). Observations of the settlement tiles throughout the experiment showed that veligers that attached appeared healthy and were growing.

## Discussion

Quagga mussels successfully completed key life history events at the lower limit of calcium concentrations. Lakes and reservoirs in the western United States that are near source mussel populations such as Lake Mead are more vulnerable to establishment than previously expected if calcium tolerance as the limiting factor is solely considered. Low calcium concentrations have been used as criteria by national and regional assessments to classify these high elevation waterbodies as “very low risk” given calcium levels ≤12 ppm (Cohen, 2007; Whittier et al., 2008). Successful life history performance of adults and veligers at 12–15 ppm calcium in our experiments, combined with the widely-acknowledged adaptability of quagga mussels to a broad range of other water quality variables such as temperature, dissolved oxygen, pH and salinity, elevates the risk of dreissenid establishment in many waterbodies of the western United States.

Results from these experimental bioassays using Lake Tahoe water indicate that low calcium waters can support mussels through key life stages (adult, veliger) and life history processes (gametogenesis and larval settlement). Adults and veligers are likely to be the stages that are transported into novel waterbodies by humans (Johnson, Ricciardi & Carlton, 2001) or secondary introductions (Bobeldyk et al., 2005). Beyond individuals surviving and actively growing, a population can only be established if vital life cycle events such as production of gametes (sperm and egg) and veliger settlement occur. In this study, we indicate using experimental assays, that mussels of various life stages transported to the lower calcium waters of western United States mountain lakes like Lake Tahoe, can successfully complete these vital life stages and events that are critical for establishment and continued recruitment.

Rather small differences in calcium concentrations (e.g., 9 vs 12 ppm) improved mussel survival, growth, and reproduction potential in our bioassay treatments. Defining lake water calcium conditions by a single value or average from a grab sample collected from the water column should be applied with caution. Calcium concentrations in lakes can exhibit temporal and spatial variability. Weathering of watershed soils and atmospheric inputs are well-documented processes that change receiving waters’ calcium concentrations (Wetzel, 2001). Differences in calcium concentrations also exist at varying depths in lakes due to abiotic (e.g., precipation of CaCO_3_) and biotic (e.g., cellular uptake) processes that vary spatially. Additional sources of calcium can come from localized inputs such as inflows, concrete structures (Cohen & Weinstein, 2001), or aggregations of bivalves (Green, 1980).

Reconsidering the vulnerability of some low calcium lakes in the western United States should be based on our experimental results and previous observations of calcium “hotspots” facilitating localized dreissenid infestations in lakes of the midwestern and eastern United States. Lake Superior (12–15 ppm Ca), the largest of the Great Lakes, has shown localized establishment of zebra mussels in several large ports since first detection in 1989 despite initial reports that observances were limited to one-time occurrences (Cohen & Weinstein, 2001). Lake Champlain (15–30 ppm Ca) and Lake George (10–12.5 ppm) in the northeastern USA represent two low calcium systems that have also undergone establishment of small populations of zebra mussels in the past 20 years (Frischer et al., 2005). Though zebra mussels appear to have lower calcium requirements based on field observations (Jones & Ricciardi, 2005), our experimental results suggests that quagga mussel populations could also be sustained, albeit perhaps at low numbers, in low calcium waters. For example, if 12 ppm were applied as the lowest calcium threshold for quagga mussel establishment, then 83 locations (52% of total analyzed) in California would be at risk versus only 32 sites if 28 ppm were applied as the calcium threshold (Cohen, 2007).

One potential bottleneck identified in the life cycle of quagga mussels in low calcium waters may occur for overwintering adults. Although tenuous because we are projecting beyond the duration of the study, the Gompertz survival curves appear to indicate low survival (99% mortality) of adult mussels after 136 days at 9 ppm and 167 days at 12 ppm. In contrast, invaded waters with calcium >15 ppm could have 50–99% survivorship beyond one year. Survival of some reproducing adults from fall to spring is crucial for population recruitment. For example, in Lake Michigan quagga mussels must survive roughly 270 to 300 days (depending on depth) to spring when environmental cues (e.g., temperature, phytoplankton) trigger spawning events (Nalepa, Fanslow & Pothoven, 2010). Habitats with calcium levels between 9 to 12 ppm in invaded waterbodies may have limited recruitment potential if seasonal environmental cues needed to trigger spawning are delayed >170 days.

Elevated natural seston concentrations may influence rates of growth, survival, and reproductive potential in the adult experiments. Adult quagga mussels exposed to water collected from Cave Rock at Lake Tahoe (lowest calcium) and from Lake Mead (highest calcium) displayed negative or negligible growth and reduced gamete production in survivors (11% and 28%, respectively). Both treatments averaged low seston biomass (Cave Rock = 1.8 µg chl *a* L^−1^, Mead = 1.2 µg chl *a* L^−1^) compared to other treatment waters (3.9–8.1 µg chl *a* L^−1^) during the 90 day experiments. Adult quagga mussels are capable of appreciable somatic growth under low food quantity conditions (Baldwin et al., 2003); however, these food-growth experiments were conducted for a much shorter amount of time (28–52 days). The long duration exposed to low food availability (90 days) likely reduced the growth and reproductive potential in adult quagga mussels in both the low and high calcium treatments. In contrast, the ample food supply in the other low calcium water (Tahoe Keys) may have provided enough “extra” calcium through dietary uptake to supplement the pool of free ion calcium in the surrounding water. High survival rates for adult zebra mussels exposed to low calcium water (10.7 ppm) for 133 days were reported from Lake George when diets were supplemented with high densities of cultured centric diatoms (10^8^ cells L^−1^ ∼ ≥10 µg chl *a* L^−1^) (Frischer et al., 2005). The estimated 20–30% of calcium demands that can be supplied by food to mollusks (Vinogradov et al., 1993) may be a key factor that allows dreissenid adults to survive at 10 to 12 ppm. It is possible that more than a third of dreissenid calcium demands may be met by food supply through direct intake, stimulation of filtration activity or a combination mechanisms (Pynnonen, 1991). Further work that isolates the influence of food quantity, food quality, and filtration activity using across a calcium gradient seems warranted to help explain what factors directly affect mussel survival, growth and reproductive potential.

Fertilization success and zygote-to-veliger survival are two important life history stages that were not specifically tested in our experimental bioassays. Previous studies of these early stages in zebra mussels reported that successful development of eggs and larvae required calcium concentrations >24 ppm (Sprung, 1987) and potentially as high as 40–60 ppm to avoid crippled larvae (Sprung, 1993). Recently, Baldwin et al. (2012) conducted bioassays using quagga mussel eggs and zygotes exposed to a gradient of calcium concentrations from waters within the St. Lawrence watershed. These experimental results showed that fertilization was successful at low calcium concentrations comparable to Lake Tahoe waters. Based on their probability functions, there could be 13% success in 9ppm and 32% success in 12ppm water. Similarly, probability functions based on bioassays by Baldwin et al. (2012) predict survival of zygotes to D-veliger at 7% and 17%. Successful fertilization and larval survival improved substantially (>50%) above 18ppm for both life history stages in their experiments. Despite these probabilities appearing relatively low, these data suggest that there is still a chance of success in low calcium waters. This early life history information considered with the extremely high fecundity of female dreissenids (e.g., millions of eggs) should lend caution to regarding these developmental stages as a dead-end for potential population establishment in low calcium waterbodies.

An important abiotic parameter besides calcium to consider when evaluating dreissenid establishment risk is the pH range in the water-body (Ramcharan, Padilla & Dodson, 1992). We did not specifically evaluate pH affects in our experimental design, however the treatment water in veliger bioassays may have approached pH values of 7 during the experimental periods. Typically the lower limit tolerances used for predicting potential distributions for zebra mussels, and assumed to be the same for quagga, is 6.5 to 7.5 (Cohen, 2005). Veligers in the Tahoe experimental treatments may have experienced additional stress if pH approached this lower limit. In this case, the veliger survivorship should have actually achieved higher success in all Tahoe treatments if treatments had attained the ambient pH levels of Lake Tahoe (>7.5).

Our experimental results obtained from bioassays of various life history stages (adults, veliger survival/growth) and events (gametogenesis, larval settlement) provide a more complete understanding of the potential establishment threat that quagga mussels pose in low calcium lakes. Other system attributes aside from water column calcium levels should be considered during assessments, such as the potential for localized “hot-spots” of calcium (e.g., dense beds of bivalves, localized inputs from streams). Several national, regional, and state risk assessments for dreissenid invasions have applied the best available field and laboratory data to help guide these risk categories (e.g., Not vulnerable, Low Risk, High Risk) (Cohen, 2007; Whittier et al., 2008). These thresholds for risk categories should not be viewed as fixed, but should be continually evaluated as new data becomes available and updated. Often the interpretation and application of these new data to evaluate risk is facilitated by expert judgment and management objectives at the state and local level. Given experimental results similar to ours, questions arise such as what outcomes in life history performance warrant adjusting the thresholds for risk categories? Past and future investments in costly inspection and decontamination activities are clearly needed to prevent introductions of quagga mussels as well as other invasive species. Ideally, if resources allow, the certainty of risk categories could be tested within each waterbody of concern and is highly recommended for systems of major ecological and economical benefit that are near these thresholds (e.g., 12 ppm Ca). Beyond water-body specific risk evaluations with experiments, major efforts should also be made to incorporate these mussel life history data (e.g., probability of egg development, veliger survival, post-settlement survival, etc.) into population models that can better inform regional water managers.

## Supplemental Information

10.7717/peerj.1276/supp-1 Supplemental Information 1 Raw data survival and growth rate analyses

## Additional Information and Declarations

### Field Study Permissions

The following information was supplied relating to field study approvals (i.e., approving body and any reference numbers): The Nevada Department of Wildlife (Scientific Collection Permit S36655).

## References

[ref-1] Baldwin BS, Carpenter M, Rury K, Woodward E (2012). Low dissolved ions may limit secondary invasion of inland waters by exotic round gobies and dreissenid mussels in North America. Biological Invasions.

[ref-2] Baldwin BS, Mayer MS, Dayton J, Pau N, Mendillo J, Sullivan M, Moore A, Ma A, Mills EL (2003). Comparative growth and feeding in zebra and quagga mussels (Dreissena polymorpha and Dreissena bugensis): implications for North American lakes (vol 59, pg 680–694, 2002). Canadian Journal of Fisheries and Aquatic Sciences.

[ref-3] Bobeldyk AM, Bossenbroek JM, Evans-White MA, Lodge DM, Lamberti GA (2005). Secondary spread of zebra mussels (Dreissena polymorpha) in coupled lake-stream systems. Ecoscience.

[ref-4] Claudi R, A, Graves AC, Taraborelli RJ, Prescott SE, Mastitsky (2012). Impact of pH on survival and settlement of dreissenid mussels. Aquatic Invasions.

[ref-5] Cohen AN (2005). A review of zebra mussel’s environmental requirements. A report for the California Department of Water Resources.

[ref-6] Cohen AN (2007). Potential Distribution of Zebra Mussels (Dreissena polymorpha) and Quagga Mussels (Dreissena bugensis) in California. Phase 1 Report—A Report for the California Department of Fish and Game.

[ref-7] Cohen AN, Weinstein A (2001). Zebra mussel’s calcium threshold and implications for its potential distribution in North America. San Francisco Estuary Institute, Oakland, CA. A report for the California Sea Grant College Program, La Jolla CA, and the Department of Energy.

[ref-8] Cox DR (1962). Renewal theory.

[ref-9] Frischer ME, McGrath BR, Hansen AS, Vescio PA, Wyllie JA, Wimbush J, Nierzwicki-Bauer SA (2005). Introduction pathways, differential survival of adult and larval zebra mussels (Dreissena polymorpha), and possible management strategies, in an adirondack lake.

[ref-10] Gompertz B (1825). On the nature of the function expressive of the law of human mortality and on a new mode of determining life contingencies. Philosophical Transactions of the Royal Society of London.

[ref-11] Green RH (1980). Role of a unionid clam population in the calcium budget of a small arctic lake. Canadian Journal of Fisheries and Aquatic Sciences.

[ref-12] Hecky RE, Smith REH, Barton DR, Guildford SJ, Taylor WD, Charlton MN, Howell T (2004). The nearshore phosphorus shunt: a consequence of ecosystem engineering by dreissenids in the Laurentian Great Lakes. Canadian Journal of Fisheries and Aquatic Sciences.

[ref-13] Horvath TG, Lamberti GA, Lodge DM, Perry WL (1996). Zebra mussel dispersal in lake-stream systems: Source–sink dynamics?. Journal of the North American Benthological Society.

[ref-14] Hosmer DW, Lemeshow S (1999). Applied Survival Analysis: Regression Model of Time to Event Data.

[ref-15] Johnson LE, Ricciardi A, Carlton JT (2001). Overland dispersal of aquatic invasive species: A risk assessment of transient recreational boating. Ecological Applications.

[ref-16] Jones LA, Ricciardi A (2005). Influence of physicochemical factors on the distribution and biomass of invasive mussels (Dreissena polymorpha and Dreissena bugensis) in the St. Lawrence River. Canadian Journal of Fisheries and Aquatic Sciences.

[ref-17] Karatayev AY, Burlakova LE, Padilla DK (1997). The effects of Dreissena polymorpha (Pallas) invasion on aquatic communities in eastern Europe. Journal of Shellfish Research.

[ref-18] Karatayev AY, Burlakova LE, Padilla DK (1998). Physical factors that limit the distribution and abundance of Dreissena polymorpha (Pall.). Journal of Shellfish Research.

[ref-19] Mackie GL, Claudi R (2013). Monitoring and Control of Macrofouling Mollusks in Fresh Water Systems.

[ref-20] Marker AF, Crowther H, C A, Gunn RJM (1980). Methanol and acetone as solvents for estimating chlorophyll-a and phaeopigments by spectrophotometry. Archives of Hydrobiology Bulletin (Ergebnisse der Limnologie).

[ref-21] Mills EL, Rosenberg G, Spidle AP, Ludyanskiy M, Pligin Y, May B (1996). A review of the biology and ecology of the quagga mussel (Dreissena bugensis), a second species of freshwater dreissenid introduced to North America. American Zoologist.

[ref-22] Nalepa TF, Melis TS, Hamill JF, Bennett GE, Coggins LG, Grams PE, Kennedy TA, Kubly DM, Ralston BE (2008). An overview of the spread, distribution, and ecological impacts of the quagga mussel, Dreissena rostriformis bugensis, with possible implications to the Colorado river system.

[ref-23] Nalepa TF, Fanslow DL, Pothoven SA (2010). Recent changes in density, biomass, recruitment, size structure, and nutritional state of Dreissena populations in southern Lake Michigan. Journal of Great Lakes Research.

[ref-24] Nalepa TF, Schloesser DW (2013). Quagga and Zebra Mussels: Biology, Impacts, and Control.

[ref-25] Pynnonen K (1991). Accumulation of 45Ca in the freshwater unionids Anodonta anatina and Unio tumidus, as influenced by water hardness, protons, and aluminum. Journal of Experimental Zoology.

[ref-26] R Core Team (2013). R: A language and environment for statistical computing.

[ref-27] Ramcharan CW, Padilla DK, Dodson SI (1992). Models to predict potential occurrence and density of the zebra mussel, Dreissena polymorpha. Canadian Journal of Fisheries and Aquatic Sciences.

[ref-28] Schneider DW, Stoeckel J a, Rehmann CR, Blodgett KD, Sparks RE, Padilla DK (2003). A developmental bottleneck in dispersing larvae: Implications for spatial population dynamics. Ecology Letters.

[ref-29] Sprung M (1987). Ecological requirements of developing Dreissena polymorpha eggs. Archiv fur Hydrobiologie.

[ref-30] Sprung M, Nalepa TF, Schloesser DW (1993). The other life: An account of present knowledge of the larval phase of Dreissena polymorpha. Zebra Mussels: Biology, Impacts, and Control.

[ref-31] Strayer DL, Hattala KA, Kahnle AW (2004). Effects of an invasive bivalve (Dreissena polymorpha) on fish in the Hudson River estuary. Canadian Journal of Fisheries and Aquatic Sciences.

[ref-32] Strickland JDH, Parsons TR (1972). A Practical Handbook of Seawater Analysis.

[ref-33] Thorp JH, Alexander JE, Cobbs GA (2002). Coping with warmer, large rivers: a field experiment on potential range expansion of northern quagga mussels (Dreissena bugensis). Freshwater Biology.

[ref-34] USEPA (2009). National Lakes assessment: a collaborative survey of the Nation’s Lakes.

[ref-35] Vinogradov GA, Smirnova NF, Sokolov VA, Bruznitsky AA, Nalepa TF, Schloesser DW (1993). Influence of chemical composition of the water on the mollusk Dreissena polymorpha. Zebra mussels: biology, impacts, and control.

[ref-36] Wetzel RG (2001). Limnology: lake and river ecosystems.

[ref-37] Whittier TR, Ringold PL, Herlihy AT, Pierson SM (2008). A calcium-based invasion risk assessment for zebra and quagga mussels (Driessena spp). Frontiers in Ecology and the Environment.

[ref-38] Wimbush J, Frischer ME, Zarzynski JW, Nierzwicki-Bauer SA (2009). Eradication of colonizing populations of zebra mussels (Dreissena polymorpha) by early detection and SCUBA removal.

[ref-39] Wright DA, SetzlerHamilton EM, Magee JA, Kennedy VS, McIninch SP (1996). Effect of salinity and temperature on survival and development of young zebra (Dreissena polymorpha) and quagga (Dreissena bugensis) mussels. Estuaries.

